# Maintenance Electroconvulsive Therapy, Developmental Regression, Depression and Catatonia in an Adolescent With Down Syndrome: A Case Report

**DOI:** 10.7759/cureus.31905

**Published:** 2022-11-26

**Authors:** Gameli Anthonio, Fayeza Malik, Armin Ghomeshi, Olga Lopez, Leonard Gralnik

**Affiliations:** 1 Psychiatry, Florida International University, Herbert Wertheim College of Medicine, Miami, USA; 2 Medicine, Florida International University, Herbert Wertheim College of Medicine, Miami, USA

**Keywords:** developmental regression, major depression disorder, electroconvulsive therapy (ect), treatment-resistant catatonia, down's syndrome

## Abstract

This is a case report involving a 22-year-old male with a past medical history of Down syndrome and major depressive disorder who, at age 16, became preoccupied with returning to an infant-like state. He experienced a gradual deterioration in his mood over a year and began to show symptoms consistent with catatonia. These symptoms included waxy flexibility, hypokinesis, decreased appetite, mutism, and altered sleep habits. Pharmacologic therapy was initiated, and the patient experienced a waxing and waning pattern of improvement and regression. Over several years, various combinations of antidepressants, benzodiazepines, and second-generation antipsychotics were attempted. The patient and his family discontinued all medications except his benzodiazepine in early 2019 and decided to try electroconvulsive therapy (ECT). After more than 100 sessions of ECT between 2019 and 2022, the patient showed notable improvement in overall mood, and his appetite and sleep completely returned to baseline. His speech, affect, and movement also improved. With ECT, the patient showed the most sustained and substantial improvement in his catatonic symptoms. ECT has been historically shown to improve these types of symptoms in catatonic patients, including those who have Down syndrome. Often, clinicians do not consider the possibility of catatonia in patients with this type of presentation, which is unfortunate as misdiagnosis leads to increased morbidity. Additionally, there has not been much discussion of the optimal length of treatment and the necessity of slowly tapered maintenance therapy in the literature. This case report illustrates how catatonia can be a major cause of developmental regression in patients with Down syndrome and offers an example of a promising management strategy for the treatment of this condition.

## Introduction

Down syndrome is one of the most common genetic disorders in the United States, affecting one in 700 live births [1]. Down syndrome (DS) or trisomy 21 arises when there is aberrant duplication of chromosome 21, either by translocation, an extra free-stranding chromosome, or mosaicism. It is classically associated with physical abnormalities such as dysmorphic facies, growth delay, abdominal wall defects, heart disease, and gastrointestinal dysfunction. There are also many comorbid psychiatric manifestations associated with Down syndrome, with intellectual disability, attention-deficit hyperactivity disorder, oppositional-defiant disorder, and mood disorders being the most common [2].

In the setting of severe, treatment-refractory mood disorders, catatonia can also arise. Catatonia is a complex neuropsychiatric syndrome typically manifesting with a combination of motor and behavioral abnormalities characterized by marked hypoactivity [3]. Catatonia manifests as a subtype of a distinct general medical or psychiatric syndrome or, in the setting of a particular disorder-not as a discrete, isolated medical condition [4]. Common signs include hypokinesis, mutism, decreased alertness, waxy flexibility, posturing, staring, echolalia, echopraxia, and excessive, purposeless movement [4]. Subtypes of catatonia include retarded catatonia, in which patients present with predominantly hypoactive symptoms, including mutism, hypokinesis, posturing, and staring, along with decreased oral intake and fecal or urinary incontinence in severe cases. Conversely, in excited catatonia, patients display excessive motor activity, restlessness, stereotyping, impulsivity, aggression, and rarely, delirium [5]. Malignant catatonia is a potentially lethal subtype characterized by marked autonomic dysfunction and fever [6]. Therefore, if it is not identified and managed promptly, catatonia can lead to increased morbidity.

Catatonia is typically evaluated with the Bush-Francis Catatonia Rating Scale (BFCRS) in both clinical and research settings. The BFCRS is the preferred rating scale for screening and diagnosis due to its reliability, validity, and short administration time [7]. It is based on descriptors of catatonia and motor signs that have been identified in the Diagnostic and Statistical Manual of Mental Disorders V (DSM V). The full BFCRS includes 23 questions used to determine the presence of symptoms commonly associated with catatonia (i.e., mutism, starting, posturing, echopraxia, rigidity, etc.). If two or more of the first 14 questions are positive, the patient meets diagnostic criteria for catatonia, and the remaining nine questions are completed to determine severity [8]. Management of catatonia typically includes treating the underlying mood disorder (if present) along with benzodiazepines and electroconvulsive therapy (ECT) to address the catatonic symptoms [5]. Additionally, other therapies are in beginning to be investigated. In a recent case report by Hart et al., a patient with DS and catatonia was treated with immunotherapy and corticosteroids and experienced near-complete resolution of catatonic symptoms [9]. 

A largescale meta-analysis showed the prevalence of catatonia to be 7.8% within the general population worldwide [10]. However, the prevalence of catatonia among psychiatric patients can be as high as 10% [7]. Further, it has been suggested that individuals with Down syndrome are at a particularly increased risk of developing catatonia. The mechanism for this phenomenon is not well understood [11]. Thus, it is important to identify and treat these patients appropriately, as catatonia has been shown to lead to clinical deterioration in Down syndrome patients [11].

In the past decade, there has been a growing scientific interest in the clinical deterioration of Down syndrome patients. In the context of Down syndrome, clinical deterioration and developmental regression usually refer to an episode of rapid functional, behavioral, and cognitive decline. Patients typically display psychiatric symptoms, including depressed mood, social withdrawal, and reduced ability to conduct the activities of daily living (ADL) [11]. Traditionally, when these symptoms arise in patients with Down syndrome, they are first attributed to organic causes such as thyroid disorders, anemia, seizure disorders, or cardiac disease. After general medical illnesses are ruled out, early-onset Alzheimer's dementia (AD) is generally thought of as the next most likely culprit. However, AD, in contrast to catatonia, is a slowly progressive, irreversible cause of the neurocognitive decline. Many patients who meet the criteria for catatonia do not receive treatment, suggesting that many clinicians do not consider the possibility of this diagnosis. This is unfortunate as misdiagnosis leads to increased morbidity [11].

Catatonia is very responsive to first-line treatment with benzodiazepines (such as lorazepam) or ECT for refractory cases [3]. A positive Lorazepam Challenge Test (administration of one to two mg of Lorazepam by mouth, intravenously, or intramuscularly resulting in rapid but temporary resolution of all symptoms) supports a diagnosis of catatonia. If the improvement is seen after the administration of lorazepam, increasing doses up to 30 mg in adults and 24 mg in adolescents are recommended with careful monitoring for sedation, respiratory compromise, and other adverse effects [12]. Patients who have no improvement with lorazepam or who require a decisive and rapid response for life-threatening conditions like malignant catatonia with autonomic dysregulation (fever, tachycardia, blood pressure changes) may also require ECT [7].

ECT is a well-established and highly effective treatment for catatonia [3]. In ECT, patients are placed under anesthesia and exposed to brief electrical pulses to the brain. Theoretically, this increases cortical blood flow to stimulate GABA activity in the brain. Patients often require multiple courses of treatment to achieve full resolution of symptoms. Even then, symptoms may recur, necessitating long-term maintenance of ECT. It is often hard to determine how the duration of ECT treatment will lead to full resolution, but predictors of favorable outcomes include young age, baseline autonomic dysregulation, and daily ECT during the first week of treatment [3].

We present the case of a 22-year-old patient with Down syndrome who displayed significant symptoms of regression and catatonia in the setting of refractory major depression following his parents’ divorce. Multiple treatment modalities were attempted for this patient, including selective serotonin inhibitors (SSRIs), benzodiazepines, antipsychotics, and ECT, with the combination of lorazepam and ECT showing the most considerable and sustained improvement. This case report provides insight into the efficacy of this combined treatment modality for patients with similar diagnoses and highlights the prevalence of catatonia as a treatable cause of regression in Down syndrome.

## Case presentation

The patient is a 22-year-old male with a past medical history of Down syndrome. He initially presented to the clinic in 2016 at 17 years old after his parents noticed a deterioration in his mood over the past year. Before his decline, the patient had a high functional status, which included giving talks at conferences with large audiences and involvement in multiple extracurricular activities. Four months before presenting to our center, there was an abrupt change in the patient’s behavior. He suddenly began avoiding speaking, and when he did speak, his voice was noticeably softer and, at times, inaudible. He began whispering, using microphones, writing, typing, and using sign language to communicate. In addition, his parents noted that he often isolated himself and began to walk very slowly. The patient also reported decreased appetite, decreased need for sleep, and having little interest or pleasure in doing things. Upon examination, he endorsed feeling confused more often and having difficulty remembering dates. He described his overall mood as feeling “down”. Stressors included the death of at least one grandparent and another relative, along with the dissolution of his parent's marriage. He attributed his feelings of sadness to this volatility in his parent's relationship. He stated his parents had been arguing before that and expressed his desire for wanting to be “a young child again, back when everything was good,” alluding to the time when his parents were together seven years before their divorce. As per his parents, the patient had become obsessive about not wanting to get old and had even started shaving his body and facial hair to appear younger. At that time, the patient denied suicidal or homicidal thoughts and denied auditory or visual hallucinations. The patient was started on 100 mg of sertraline daily, which provided moderate transient symptom improvement but did not return him to his baseline.

The patient continued to show symptoms of catatonia, and the BFCRS scale was conducted to characterize his condition. He scored 17 on the BFCRS, which was consistent with moderate to severe catatonia. Upon further examination, the patient displayed poor eye contact and had a blunted affect. He denied any use of alcohol, tobacco, or illicit drugs. At times, history was obtained from the parents due to mutism. In addition, a full neurologic workup, including MRI and EEG, was unremarkable. Over the next year, the patient continued taking sertraline 100 mg once a day for mood and anxiety and lorazepam, one mg twice a day, was added and eventually increased to a maximum dose of two mg four times a day to treat his symptoms of catatonia. With this medical regimen, the patient’s waxy flexibility persisted, but his speech and hypokinesis showed improvement. Despite this moderate response, the patient continued to report a low mood and anhedonia. He was tolerating both the sertraline and the lorazepam well, never showing signs of sedation, but felt that “the medicine wasn’t working” and endorsed continuing to feel “sad.” The patient was thus switched from sertraline 100 mg once daily to fluoxetine 10 mg once daily to treat his residual depressive symptoms. Six months later, the patient’s symptoms persisted, and his fluoxetine dose was increased to 20 mg once daily.

**Figure 1:**
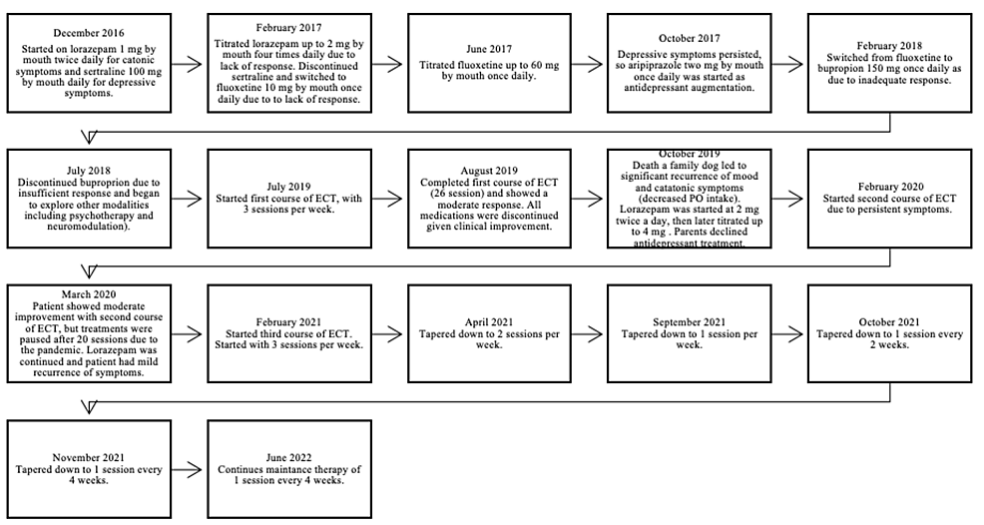
Treatment Regimen Progression

Through 2016 and 2017, the patient demonstrated minimal improvement (Figure 1). He began talking spontaneously and more often and displayed normal movement and posture. In mid-2017, his dose of fluoxetine was titrated up to 60 mg one time daily; however, he still reported sadness, continued to whisper while talking and remained preoccupied with reverting to a younger age. In late 2017, aripiprazole two mg once daily was added to his regimen (lorazepam two mg four times a day and fluoxetine 60 mg once daily) as augmentation and provided some improvement in his depressive symptoms, but his signs of delusional thinking remained. The patient was switched from fluoxetine to bupropion 150 mg once daily in early 2018 due to these residual depressive symptoms. In late 2018, the patient and his caregivers opted to discontinue all antidepressants and continue on lorazepam two mg four times daily and aripiprazole two mg once daily while exploring options such as psychotherapy, transcranial magnetic stimulation (TMS), ECT, or a combination of these modalities. In mid-2019, due to the patient’s persistent feelings of sadness, mutism, and hypokinesis, the patient and his family started ECT. First course of ECT, with a total of 26 sessions, resulted in some improvement. All medications were discontinued as a result. Following the death of his family’s dog, the patient’s depression recurred. He was not speaking, only communicated through text, and endorsed frequent tearfulness, decreased appetite with a 14-pound weight loss and low mood. Lorazepam was restarted and titrated up to two mg four times a day. The patients and his parents preferred to not restart antidepressant therapy due to lack of response during previous trial. Patient started a brief second course of ECT in February 2020. Treatment consisted of approximately 20 sessions and was showing some benefit but was paused due to the COVID-19 pandemic. His symptoms subsequently worsened after treatment was halted. In early 2021, the third course of ECT was started and has proved to be the most beneficial. The regimen began with three treatments per week and has now been tapered down to one maintenance treatment every four weeks in an attempt to sustain his improvement. The patient received more than 100 sessions of ECT between 2019 and 2022. Additionally, lorazepam has been continued, consistent with treatment recommendations in the literature [11].

Upon evaluation after initiating the second course of ECT, the patient has shown his most marked improvement since the onset of treatment. His speech has returned to a normal volume with complete sentences, and his movements are no longer grossly hypokinetic. His appetite and sleep have returned to his previous baseline. His behavior no longer shows evidence of a fixation on infancy. Additionally, he now smiles frequently and reports an overall improved mood, stating he is feeling “a lot better.” 

## Discussion

Catatonia has been historically associated with multiple psychiatric conditions, including mood disorders such as major depressive disorder. Recently, however, the condition has become an object of increased interest among patients diagnosed with Down syndrome [11]. Due to its wide range of symptoms and clinical presentation, catatonia has traditionally been overlooked or misattributed in these patients, but case series have shown a link between catatonia and Down syndrome patients [13]. Various treatment options have been implemented to decrease symptoms and improve outcomes in these patients. Because catatonia is a cluster disorder that includes motor dysfunction, mood changes, and cognitive decline, a combination of pharmacological options and ECT is often used. ECT specifically has been shown to improve symptoms in Down syndrome patients presenting with catatonia [14].

Overall, this patient’s clinical presentation was very similar to other cases of catatonia in Down syndrome reported in the literature. He displayed a rapid decline in mood and social engagement, which is consistent with other reported cases in which patients quickly deteriorate over months [15]. Additionally, the patient showed classic catatonia symptoms such as social withdrawal, mutism, hypokinesis, decreased need for sleep, and feeling “down.” The patient displayed thought content concerning delusional thinking (i.e., preoccupation with infancy) but did not report any overt auditory and visual hallucinations, which are sometimes seen in catatonic patients [15].

Similar to the treatment approach used in other patients, a combination of pharmacological therapies, including benzodiazepines, SSRIs, antipsychotics and ECT, were used in this case. According to the literature, benzodiazepines are the first-line treatment for catatonia and offer an additional benefit as a diagnostic probe [12]. Although response rates in ECT have not been systematically studied, its efficacy and benefits have been reported in hundreds of case reports [16]. When the patient’s symptoms of depression, delayed movement and mutism proved to be refractory to pharmacotherapy alone, the patient and his parents agreed to ECT therapy. Historically, ECT has been shown to improve these types of symptoms in catatonic patients, including those who have Down syndrome [14]. There is less data available in the literature about the optimal approach to ECT in catatonic patients with Down syndrome. Our patient, in this case, displayed insufficient response to treatment until ECT was initiated and experienced frequent recurrence of symptoms when ECT was stopped abruptly without a sufficient taper. This case shows that without a prolonged and slowly tapered ECT regimen, relapse is likely. This patient’s condition improved substantially after more than 100 ECT sessions, with near full resolution of motor and speech symptoms and increased social engagement and elevated mood. 

## Conclusions

Catatonia is a major cause of developmental regression in Down syndrome patients, as this case illustrated. Because catatonia is both treatable and potentially life-threatening in severe cases, future studies should take a systematic approach to investigate more definitive treatment protocols for ECT use and other therapeutic modalities. For instance, the use of maintenance therapy has been widely reported, but the lack of research on the duration and frequency of treatment makes it difficult to create official guidelines, especially for specific patient populations. We found that a long period of maintenance treatment was necessary to prevent relapse of catatonia and regression in this patient, as evidenced by his significant relapse after the first course of ECT ended, despite continuing lorazepam. The combination of ECT with other medications has been used, but future studies can help elucidate information that will allow for the optimization of treatment regimens based on unique symptom presentations in different patients. Additional modalities like immunotherapy and systemic corticosteroids should also be investigated to further evaluate their utility when combined with standard pharmacological therapy and ECT, especially in the context of Down syndrome. Because catatonia can present in a multitude of medical and psychiatric conditions, clinicians often face challenges in choosing a therapeutic approach. As more high-quality data is produced, the long-term effects of catatonia management can be highlighted so patients and their families can make better-informed decisions regarding treatment.
